# Comparing the Effects of Road, Railway, and Aircraft Noise on Sleep: Exposure–Response Relationships from Pooled Data of Three Laboratory Studies

**DOI:** 10.3390/ijerph16061073

**Published:** 2019-03-26

**Authors:** Eva-Maria Elmenhorst, Barbara Griefahn, Vinzent Rolny, Mathias Basner

**Affiliations:** 1 Institute of Aerospace Medicine, German Aerospace Centre (DLR), 51170 Cologne, Germany; vinzent.rolny@roche.com (V.R.); basner@pennmedicine.upenn.edu (M.B.); 2 Institute for Occupational and Social Medicine, Medical Faculty, RWTH Aachen University, 52074 Aachen, Germany; 3 Leibniz Research Centre for Working Environment and Human Factors, 44139 Dortmund, Germany; barbara.griefahn@udo.edu; 4 Unit for Experimental Psychiatry, Division of Sleep and Chronobiology, Department of Psychiatry, Perelman School of Medicine, University of Pennsylvania, Philadelphia, PA 19104, USA

**Keywords:** railway noise, aircraft noise, road traffic noise, awakening, sleep, noise legislation, exposure–response curve

## Abstract

Objectives: Air, road, and railway traffic, the three major sources of traffic noise, have been reported to differently impact on annoyance. However, these findings may not be transferable to physiological reactions during sleep which are considered to decrease nighttime recovery and might mediate long-term negative health effects. Studies on awakenings from sleep indicate that railway noise, while having the least impact on annoyance, may have the most disturbing properties on sleep compared to aircraft noise. This study presents a comparison between the three major traffic modes and their probability to cause awakenings. In combining acoustical and polysomnographical data from three laboratory studies sample size and generalizability of the findings were increased. Methods: Data from three laboratory studies were pooled, conducted at two sites in Germany (German Aerospace Center, Cologne, and Leibniz Research Centre for Working Environment and Human Factors, Dortmund). In total, the impact of 109,836 noise events on polysomnographically assessed awakenings was analyzed in 237 subjects using a random intercept logistic regression model. Results: The best model fit according to the Akaike Information Criterion (AIC) included different acoustical and sleep parameters. After adjusting for these moderators results showed that the probability to wake up from equal maximum A-weighted sound pressure levels (SPL) increased in the order aircraft < road < railway noise, the awakening probability from road and railway noise being not significantly different (*p* = 0.988). At 70 dB SPL, it was more than 7% less probable to wake up due to aircraft noise than due to railway noise. Conclusions: The three major traffic noise sources differ in their impact on sleep. The order with which their impact increased was inversed compared to the order that was found in annoyance surveys. It is thus important to choose the correct concept for noise legislation, i.e., physiological sleep metrics in addition to noise annoyance for nighttime noise protection.

## 1. Introduction

Disruptions of the physiological sleep process were found to cause the greatest burden of disease from environmental noise [1,2]. Short and disturbed sleep has been associated with increased cardiometabolic morbidity and mortality [3,4]. So, long-term negative health effects of traffic noise [5,6,7] might be mediated via sleep disruptions. Secondary reactions to noise like daytime sleepiness and performance impairments [8,9,10,11,12], and subjective daytime annoyance have been subject to intense research activities [13,14,15,16]. Annoyance has been reported to vary in degree depending on traffic modes, i.e., residents feel most annoyed by aircraft noise, and least by railway noise with road traffic noise ranging in between [17,18] when exposed to an equal A-weighted sound pressure level (SPL). Results of these and similar annoyance surveys led to higher limits in noise legislation for railway noise in some European countries. Investigations in six European countries have shown that annoyance due to road traffic noise is still comparable to the level reported in Miedema and Vos [18], whereas annoyance due to aircraft noise has increased [19]. However, in a laboratory study that examined the impact of air, road, and railway noise on polysomnographically assessed sleep structure in a systematic approach, this order was inversed and polysomnographically assessed sleep disturbances increased in the order air < road < railway noise [20].

Arousals and awakenings during sleep are short and often not consciously perceived; the macrostructure of sleep is seldom significantly altered by noise [20,21,22], and subjective ratings of sleep are difficult to interpret. Analyses that show an association between the single noise event and a subsequent awakening in the sleep electroencephalogram (EEG) allow the calculation of noise exposure–response relationships. However, studies on noise effects using polysomnography—the gold standard for recording sleep—analyzing the data in an event-related approach are rare and sample sizes have been small because of the cost of the methodology in terms of instrumentation, time, and personnel [23]. In the present analyses we pooled data from different laboratory studies that were concerned with the effects of nocturnal road, railway, and aircraft noise on polysomnographically recorded awakenings during sleep. The pooled database allowed for the development of exposure–response relationships based on a large subject sample from two laboratories, thus increasing the generalizability of the findings.

## 2. Material and Methods

### 2.1. Participants

Datasets of three laboratory studies were used:

(1) STRAIN (Study on human specific response to aircraft noise) study at the German Aerospace Center, Cologne: 112 participants (65 female, 47 male) with an average age of 38.1 years ± 12.9 (SD) ranging from 19 to 65 years.

(2) AIRORA (Effects of air, road and rail traffic noise) study at the German Aerospace Center, Cologne: 72 participants (40 female, 32 male) enrolled in the study with a mean age of 40.3 years ± 13.4 (SD) and an age range from 18 to 71 years.

(3) IfADo (Leibniz-Institut für Arbeitsforschung an der TU Dortmund) study at the Leibniz Research Centre for Working Environment and Human Factors, Dortmund: 53 participants (26 female, 27 male) were examined. The age averaged 23.4 years ± 2.3 (SD) with a range from 20 to 29 years.

Subjects were selected in a multilevel selection process concerning their physical and psychological health with special focus on exclusion of participants with sleep or circadian disorders. For these purposes, in the STRAIN and AIRORA studies, self-designed questionnaires asked for medical history, symptoms indicative of unidentified diseases (such as snoring or excessive daytime sleepiness in, e.g., chronic sleep apnea or restless legs syndrome), sleep–wake rhythms, and shift work. The Freiburger Persoenlichkeits Inventar [24] was used for psychological screening. A medical check by a physician ensured good physical health. During one night we monitored participants’ heart rate and oxygen saturation at home prior to the laboratory stay to check for sleep-related breathing disorders. The first night at the sleep laboratory served as acclimatization to the laboratory environment and to the polysomnographic equipment. Trained personnel checked this night at the sleep laboratory for signs of sleep abnormalities, especially for oxygen desaturation, abnormal movements of chest or abdomen, signs of snoring, or sleep onset rapid eye movement (REM) sleep. For the detection of periodic leg movements or restless legs symptoms we relied on participant self-reporting. In the IfADo study, the same medical self-report was used. Participants had a normal hearing threshold according to their age which was verified with audiometry. Descriptions of the selection process for each study have been reported previously [20,21,25,26].

All subjects signed an informed consent according to the Declaration of Helsinki [27] and were reimbursed for participation. The STRAIN and AIRORA studies were approved by the Ethics Committee of the North Rhine Medical Board, the IfADo study was approved by the local Ethics Committee of the Institute. All datasets were anonymized for this joined work.

### 2.2. Design and Acoustics

(1) STRAIN study: Participants stayed in the sleep laboratory for 13 nights and were exposed to aircraft noise during nine consecutive nights (eight hours sleep opportunity). The first two and the last two nights were free from noise (only the second night included in the analyses as noise-free baseline night). Noise load was randomly changed in each exposure night concerning number (4, 8, 16, 32, 64, or 128 events per night) and maximum A-weighted SPL of the noise event using fast time window (between 45 to 80 dB/5 dB steps). Sixteen different noise events from aircraft had been recorded in the vicinity of Duesseldorf airport and were repeatedly played back during the night to generate these noise patterns. The A-weighted SPL (L_Aeq_T, which is calculated by integrating the sound energy of all noise events in the time T given) ranged between ≤ 30 dB under noise-free conditions to 57 dB in noisy conditions. The acoustical design has been previously reported in detail [10].

(2) AIRORA study: Participants stayed in the sleep laboratory for 11 consecutive nights. Nights 1 and 11 were noise-free (not included in the analyses), the nights in between were regarded as noise exposure nights including one noise-free night. Noise from three major traffic noise sources—air, road, and railway—was played back during the night. Noise intensity varied as follows; 0, 40, 80, or 120 noise events per night with maximum A-weighted SPL of the noise event using fast time window between 45 to 65 dB (5 dB steps). L_Aeq_T of exposure nights ranged from ≤ 30 dB under noise-free conditions to 43.3 dB. Details regarding the acoustical design of the AIRORA study have been published elsewhere [20].

(3) IfADo study: The study consisted of three similarly designed experiments (E). After an adaptation night the participants slept for 3 (E1) or 2 (E2, E3) consecutive weeks four consecutive nights each week in the laboratory. The four nights each week consisted of a randomized sequence of one quiet and three noisy nights with different acoustic conditions. A pink noise with a L_Aeq_T of 32 dB and of 28 dB was delivered throughout the quiet nights in E1 and in E2/E3, respectively.

E1: Twenty-four participants were with permuted weekly changes exposed to aircraft, to road or to railway noise. The three acoustic conditions consisted of three L_Aeq_T, namely, 39, 44, or 50 dB, that were achieved with a total number of 195 flyovers (maximum SPL varying between 46–77 dB), 261 passages of road vehicles (maximum SPL 46–74 dB), and 172 train passages (maximum SPL 45-74 dB), respectively. All maximum SPL of the noise event used fast time window and were A-weighted. To simulate a realistic scenario, the number of events decreased from 23:00 to 01:00 h and rose again from 04:00 to 07:00 h.

E2: Two groups of 8 participants each were exposed to either road or railway noise. The same noises and scenarios used in E1 were with permuted weekly changes applied in the identical version or with damped lower frequencies (<200 Hz by 12 dB).

E3: Three groups of 8 participants each (8 early sleepers: 22:00–06:00 h; 8 normal sleepers: 23:00–07:00 h; 8 late sleepers 24:00–08:00 h) were exposed with permuted weekly changes to road and to railway noise. The three acoustic conditions each week were characterized by 3 traffic curfews, i.e., 4 h from 23:00 to 3:00 h or from 3:00 to 7:00 h or 6 h from 23:00 to 5:00 h. For both 4-h curfews the number of passages was in the remaining four hours 145 for road vehicles and 94 for trains with an increasing number of vehicles after the initial and a decreasing number of vehicles before the final curfew. Due to the varying bedtimes and the curfews the L_Aeq_T varied from 39.4 to 41.7 dB with maximum SPL varying from 56 to 68 dB. The acoustic load for those who went to bed earlier or later the number of passages in the initial and the final hours (22:00–23:00 h, 07:00–08:00 h) were 48 for road and 30 for rail vehicles, respectively. For the 6-h curfew the number of passages was 124 for road and 80 for rail vehicles from 05:00 to 07:00 h. The acoustic load for early and late sleepers the number of passages during the initial and the final hours (22:00–23:00 h, 07:00–08:00 h) was 62 and 40 passages for road and rail vehicles, respectively.

After wake-up the participants rated their sleep quality, performed performance tests, and then left the institute.

From the described datasets of the three studies, in total, 109,836 noise events were available for the present analyses (Table 1).

In the following, the highest point of the sound level time course is defined as maximum A-weighted SPL of a given noise event using fast time constant [dB], and the steepest slope of the event curve as rise time of the maximum A-weighted SPL of a noise event (Tr [dB/s]).

### 2.3. Polysomnography

STRAIN and AIRORA studies: Participants’ polysomnograms were recorded including the EEG (C3, C4, F4, A1, and A2), the electrooculogram (EOG1 and EOG2), the electromyogram (EMG), electrocardiograms (ECG), respiratory movements of thorax and abdomen, and finger pulses. In the first night, participants had a thermistor that registered airflow at mouth and nose.

IfADo study: The polysomnogram (C3, C4, EOG1, EOG2, EMG, and ECG derived against A1) was recorded throughout all nights, from 23:00 to 07:00 h from all participants in E1 and E2, and from the normal sleepers in E3. The recording period was one hour earlier and later for early and late sleepers in E3.

Sleep epochs were analyzed according to standard criteria [28].

### 2.4. Statistics

The present paper focuses on the development of exposure–response relationships between aircraft, railway, and road traffic noise and awakenings from sleep. Since sleep stage S1 is a typical marker for sleep fragmentation and believed not to contribute to the recuperative effects of sleep, sleep stage changes from any other sleep stage to stage wake or S1 were considered as relevant awakening and are referred to as awakenings throughout the text. A detailed description of the event-related noise–sleep analyses is presented in [29,30].

By pooling the datasets of the three studies analyses are based on a total of 237 participants and 109,836 noise events thereby increasing variability between noise events and participants. Thus, event-related analyses of noise on awakenings have a higher generalizability compared to analyses of only a single dataset. Models based on pooled datasets have a higher ability to explain differences between the major traffic noise sources than the comparison of three separate models could offer. However, before pooling the data it must be granted that the single studies present overall consistent results so that biasing effects caused by the different study environments and populations can be regarded as negligible.

Therefore, in a first step, descriptive and inferential statistical analyses were calculated for the datasets separately. Event-related analyses between noise events and awakenings were computed using random intercept mixed logistic regression models (R 2.9., package glmmML version 0.81, The R Foundation, Vienna, Austria). In an automatic stepwise selection process variable selection for modelling was performed [31]; i.e., beginning with a simple intercept model, variables were tested separately and in combination until the best model fit was achieved according to the Akaike Information Criterion (AIC) [32]. The analyses aimed at comparing the different studies based on the magnitude of variable coefficients. Therefore, starting from the three separate best-fit models, variables were selected that were present in at least two of the three models in order to calculate again three different regression models that were now founded on the same set of variables. Since the number of noise events per night has a strong impact on awakening probability, the variable ‘number of noises per night’ was added to the model in order to take the different numbers of noise events per night in the different studies into account even though this variable was a significant moderator in the STRAIN and IfADo individual models only. Likewise, an interaction term for the current ‘noise number * maximum SPL’ and for ‘noise source * maximum SPL’ was included.

In a second step, the three datasets were pooled. As explained before, an event-related analysis between noise events and awakenings was calculated using a random intercept mixed logistic regression model (R 2.9., package glmmML version 0.81) that was now based on the pooled dataset. Again, a variable selection was performed to gain the model with the best fit according to AIC using the automatic stepwise selection process. The variables ‘number of noises per night’ as well as the interaction terms ‘noise number * maximum SPL’ and ‘noise source * maximum SPL’ were added. The candidate variables did not show collinearity (Pearson correlation: all <0.7, variance inflation factor all ≤4.0 except for ‘noise number’ = 5.2).

We modeled our dataset using a random intercept logistic regression and allowed for nonlinearity of the variables which can vary from first to third-degree polynomials. We only focused on that part of the exposure–response curve that lies within the range of the actually measured acoustical data.

Significance level was set at α < 0.05. If not otherwise mentioned values in the text are given as mean ± standard error. The standard error of the estimated mean in the presented statistical models serves as measure of uncertainty of the sampling distribution [33].

## 3. Results

### 3.1. Descriptive and Inference Statistical Results Comparing the Three Datasets

Concerning the number of noise events, the IfADo study presented twice more noise events than the STRAIN and AIRORA studies and had, thus, more weight. However, comparing the two study centers, the number of noise events was almost equal.

Table 2 outlines the results of the three regression analyses performed separately for the three datasets based on a common variable set. Noise events in the IfADo study showed slightly less impact on awakening probability compared to the STRAIN and AIRORA studies. Causes may be the higher number of noise events in the IfADo study which decreased the time interval between stimuli so that awakening probability decreased in a compensatory response, the selection of noise events itself, random differences in study populations, or effects from the varying laboratory environments. However, variable coefficients—especially of the acoustical variables for the traffic noise sources—were reasonably similar so that a valid comparison in a pooled dataset could be computed.

### 3.2. Event Related Analysis of the Pooled Dataset

**Figure 1:**
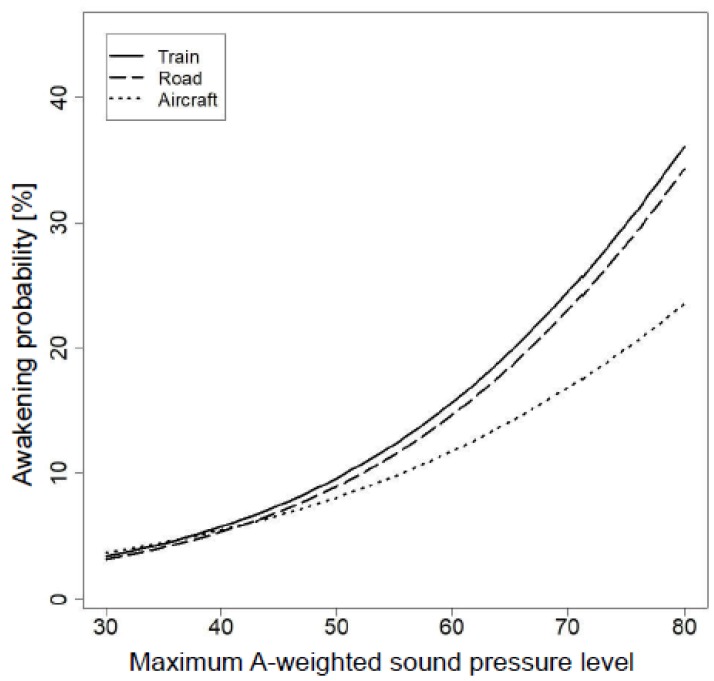
Ranking of the probability for sleep stage changes to awake and S1 due to air, road, and railway noise depending on the maximum A-weighted SPL of the noise event. Note: Exposure–response relationships based on the random effects multivariable logistic regression model presented in Table 3. Assumptions: prior sleep stage = S2; elapsed sleep time = 601 (middle of the second half of the night); elapsed sleep time in the same sleep stage (before the noise event began) = 10 epochs (median); noise-free interval (between noise events) = 3.14 min (median); Tr = 5.8 dB/s (median); noise duration = 20 s (median); noise number = 55 (median); number of noises per night = 128 (median); maximum A-weighted SPL (fast time window) at the sleeper’s ear.

The regression model with the best fit for the pooled dataset is presented in Table 3. Maximum A-weighted SPL and Tr proved to be highly significant acoustical predictors for awakenings (road traffic noise served as reference category). The significant interaction between maximum A-weighted SPL and aircraft noise indicates that the slope of the exposure–response function was less steep for aircraft noise relative to road traffic noise (Figure 1). No difference in slope was found for railway noise relative to road noise. At a maximum A-weighted SPL of 70 dB it was more than 7% less probable to wake up due to aircraft noise than due to railway noise (Figure 1).

Moreover, the probability for awakenings increased with the number of noise events per night and the longer a noise-free interval lasted. Parameters of sleep itself are furthermore important predictors: the awakening probability increased with time spent asleep, but decreased with time elapsed in the same sleep stage before the noise event occurred. Awakenings from deep sleep (S3/S4) and REM sleep were less probable as compared to the most prevalent light sleep stage S2 which served as reference for computation in this model. There was no difference in the impact of the maximum A-weighted SPL on awakening probability between studies as specified by the nonsignificant interaction between maximum A-weighted SPL and study indicator variables. The included variables seem to explain most of the design differences between the studies.

## 4. Discussion

This paper presents analyses on the effects of noise on awakenings from sleep in a pooled dataset of three large laboratory studies including 237 participants and 109,836 noise events. The event-related approach enabled the direct comparison of the impact of the three major traffic noise sources—air, road, and railway noise—on polysomnographically assessed awakening probability from sleep in an exposure–response curve. Results indicate that different traffic noise sources induce different awakening probabilities even at equal maximum A-weighted SPL and even after adjusting for acoustical parameters like Tr, number, and duration of noise events, as well as physiological parameters like current sleep stage, elapsed sleep time, and elapsed sleep time in the same sleep stage. At equal maximum A-weighted SPL the awakening probability due to the three traffic noise sources increased in the order aircraft < road < railway noise. These findings support former results from our field studies that also indicated a higher awakening probability due to railway noise in comparison to aircraft noise [9] as well as outcomes on sleep continuity [20,26].

Sleep pressure builds up during wake time and decreases with the deep sleep episodes in the first hours of the night [34]. Therefore, it is physiologically plausible that awakening probability increases with elapsed sleep time. Approximately 50% of the sleep episode humans stay in the light sleep stage S2 which is prominent in the second half of the night and from which they can be awakened easily. So, regarding physiologically based protection concepts, it is a conservative approach to adjust awakening probability for this light sleep stage and for the second half of the night [29]. The awakening probability from deep sleep and REM sleep, in contrast, is decreased in the joined model. Regarding the comparison of the different studies in separate models, REM sleep awakening probability was decreased in two of the three studies. In research on noise effects, increased [29] as well as decreased [9,35,36] awakening probabilities from REM sleep have been reported so that the picture has been equivocal. Differences in the observed awakening thresholds might be explained by noise events that coincide with tonic in contrast to phasic REM episodes [37]. In addition, the brain during REM sleep seems to have a decreased ability to distinguish between different stimulus types. A more homogeneous and monotonic arousal pattern has been reported comparing different sound sources during REM sleep in contrast to N2 or N3 sleep [38].

Recently, it has been shown that the susceptibility to noise induced awakenings or arousals is highly variable among individuals [39]. The nonlinear mixed-effect models take the clustered nature of the data (multiple measurements in a single participant) into account and specifically estimate the response variability between participants via the random intercept. The exposure–response functions represent an individual with average noise susceptibility. For noise legislation, the susceptible groups’ need for special protection should be kept in mind. It has been shown that residents around airports of higher age are more likely to be hospitalized due to cardiovascular problems [7].

As we have hypothesized before, our results further support the notion that physiological, unconscious reactions during sleep are different from psychological, conscious reactions during wakefulness, and cannot be predicted from annoyance surveys [17,18] since the impact of the traffic modes on annoyance shows an inversed ranking [9]. This ranking has also been confirmed in the AIRORA study [8]. In a survey more residents had been bothered, annoyed or disturbed from aircraft noise than from road traffic noise in the last 12 months even though noise exposure with regard to nightly L_Aeq_ and maximum A-weighted SPL of noise events had been similar [40]. Thirty-seven percent of these respondents explicitly stated that aircraft noise interfered with their sleep, whereas only 27.5% of respondents were disturbed by road traffic noise during sleep. Interestingly, a higher percentage of residents reported being bothered, disturbed or annoyed than being affected during sleep. Another field study pointed out that long-term annoyance seems differently mediated than short-term annoyance of the previous night. While 54% of participants were highly annoyed long-term by railway noise, 88% of these residents felt not or little annoyed in the morning by railway noise during the previous night [41]. Frei et al. [42] have likewise shown recently that objective sleep quality and noise annoyance are not related. Subjective sleep quality, on the contrary, proved to be mediated by noise annoyance [14,42,43]. In comparison to the EU standard curves, annoyance due to aircraft noise in contrast to road traffic noise has even increased in the past decades [19]. It is thus important to choose the correct concept for noise legislation; that is, physiological metrics from sleep in addition to noise annoyance for nighttime noise protection concepts. One such noise protection concept has been implemented at the German Airport Leipzig/Halle which is a night freight hub. The concept is based on exposure–response relationships between aircraft noise events and awakening probabilities as well as on sleep characteristics that were derived from laboratory and field investigations of aircraft noise effects on residents’ sleep. Mainly, probabilities of awakening due to aircraft noise events with certain maximum A-weighted SPL in comparison to spontaneous awakening probabilities were examined regarding the probability to recall an awakening in the morning and times that participants needed to fall asleep again. The three key elements of the protection concept are that (1) less than one additional awakening per night should be induced by aircraft noise as an annual average, (2) awakenings should not be recalled in the morning, and (3) aircraft noise should not interfere with the process of falling asleep again (detailed descriptions of the noise protection concept have been published elsewhere [29,44]). In this way physiological reactions during sleep might be minimized. Findings from laboratory studies in humans and animals indicate that nocturnal noise exposure induces some adverse metabolic [45] and vascular [46,47] reactions already in the short-term. These alterations might form the first steps on the way to excess cardiometabolic morbidity and mortality which have been linked to long-term traffic noise exposure [5,6]. The possible risk for negative health consequences underlines the importance of noise mitigation procedures that protect sleep specifically. During daytime, residents are differentially annoyed depending on the traffic noise source. Daytime noise protection concepts should consequently focus on exposure to the most annoying traffic mode.

Further characteristics of noise from the different traffic noise sources might explain the differences between reaction probabilities. Basner et al. [20] suggested that the spectral composition of the noise plays an important role in awakening probability. Especially high frequencies that are filtered for aircraft noise via the atmosphere were found to explain differences in awakening probability between traffic noise sources [20]. Recently, it has also been shown for railway noise that high frequency components are more likely to induce event-related arousals and increases in heart rate than low frequency events [48]. The fluctuations in freight train sounds as well as its sharpness have also been found to have an impact [49]. Recently, vibrations of traffic passing have been shown to be of importance [50].

## 5. Limitations

All polysomnographic scoring was performed according to standard criteria by trained personnel; however, the interrater variability is known to be high among different sleep laboratories. Also the use of different EEG software might have added to differences in scoring. In our case, analyses of interrater variability between the IfADo, the German Aerospace Center, and University of Giessen and Marburg have shown that the overall agreement had a kappa of 0.719, with excellent agreement in 38% and good agreement in 62% of cases [51].

The effects of noise on arousal probability were not available for the whole dataset, so that this marker of sleep instability could not be included in the presented analyses.

Physiological reactions due to noise have been reported to be stronger in laboratory compared to field settings [52,53,54,55]. However, these studies did not investigate whole night parameters or awakening duration. Although magnitudes of effects may differ, the rank order will likely not be affected. At least for aircraft and railway noise the same order has been found in the field [9]. A next step will be to complement the available field data on aircraft noise and railway noise with field data on road traffic noise, so that exposure–response relationships with higher ecological validity comparing the impact of the three major traffic modes on sleep can be calculated.

Including participants of all adult age groups likely increased the heterogeneity of reaction probabilities to noise in the sample as arousal propensity increases with age. The advantage is that a larger spectrum of characteristics of a normal population contributes to the derived exposure–response functions.

The subject sample chosen was healthy. People at risk, i.e., children, older participants, diseased people, and those suffering from sleep disorders or circadian misalignment, may be differently impacted by noise.

## 6. Conclusions

The awakening probability from sleep differs between the three major traffic noise sources—air, road, and railway noise. Even at equal maximum A-weighted SPL the probability to wake up increases from air to road to railway noise. This difference is persistent even if a variety of sleep parameters and acoustical parameters are taken into account. Considering noise protection concepts it still needs to be explored which features of the noise events are responsible for the differences in reaction probabilities that could be optimized [20]. The found rank order of traffic modes for awakenings from sleep is inversed in comparison to that reported for long-term annoyance. Thus, it is important to choose the correct concept for noise legislation; that is, physiological sleep metrics in addition to noise annoyance for nighttime noise protection and long-term annoyance for daytime noise protection.

## Figures and Tables

**Table 1:** Number of participants and noise events used for analyses from the three major traffic noise sources that were played back in the three different studies.

Category	STRAIN Study	AIRORA Study	IfADo Study	Total Number
Number of participants	112	72	53	237
Number of road noise events	-	9908	25,739	35,647
Number of railway noise events	-	10,014	17,666	27,680
Number of aircraft noise events	25,479	9741	11,289	46,509
Total number of noise events	25,479	29,663	54,694	109,836

**Table 2:** Separate models for the three studies: Random intercept multivariable logistic regression models concerning the relation of noise and the probability of sleep stage changes to awake/S1.

Variable	STRAIN Study		IfADo Study		AIRORA Study	
	Coefficient	*p*-Value	Coefficient	*p*-Value	Coefficient	*p*-Value
Intercept	−6.3850	<0.001	−8.1111	<0.001	−7.7395	<0.001
Maximum SPL (aircraft)	0.0473	<0.001	0.0312	<0.001	0.0421	<0.001
Maximum SPL * road	×	×	0.0196	0.002	0.0203	0.001
Maximum SPL * railway	×	×	0.0173	0.007	0.0260	<0.001
Tr (aircraft)	0.0241	0.001	0.2545	<0.001	0.0331	0.001
Tr (road)	×	×	−0.2958	<0.001	−0.0126	0.417
Tr (railway)	×	×	−0.2272	0.001	−0.0292	0.065
Railway (indicator)	×	×	−0.6031	0.054	−0.7669	0.041
Aircraft (indicator)	×	×	−0.3934	0.213	−0.9380	0.013
Noise-free interval	0.0075	<0.001	0.0077	<0.001	0.0120	0.020
Elapsed time in sleep stage	−0.0108	<0.001	−0.0022	0.034	−0.0147	<0.001
Noise duration	0.0014	<0.001	0.0019	<0.001	0.0020	<0.001
Noise number	0.0239	<0.001	−0.0013	0.543	−0.0022	0.710
Number of noise events per night	−0.0041	<0.001	0.0024	0.001	−0.0002	0.870
Maximum SPL * noise number	−0.0005	<0.001	−0.00004	0.305	−0.0001	0.575
Sleep stage S3 + S4	−0.8414	<0.001	−0.3752	<0.001	−0.7607	<0.001
REM sleep	−0.4813	<0.001	0.0292	0.407	−0.2957	<0.001

Maximum SPL = maximum A-weighted sound pressure level (SPL) of noise events [dB] from the three major traffic noise sources aircraft, road, and railway traffic; Tr = steepest slope of the event curve as rise time of the maximum A-weighted SPL of a noise event [dB/s]; noise-free interval = noise-free interval between two noise events [min]; elapsed time in sleep stages = cumulative time that participants spent in a certain sleep stage before onset of a noise event [epochs]; noise duration = duration of a noise event [s]; noise number = current number of a noise event (noise events were counted consecutively); number of noise events per night = total number of noise events per night; sleep stage S3 + S4 = deep sleep; REM sleep = rapid eye movement sleep.

**Table 3:** Random intercept multivariable logistic regression model concerning the relation of road, railway, and aircraft noise events and the probability of sleep stage changes to awake/S1.

Variable	Regression Coefficient (Standard Error)	*z*-Value	*p*-Value
Intercept	−5.5220 (0.2584)	−21.37	<0.001
Maximum SPL (road)	0.0635 (0.0038)	16.87	<0.001
Maximum SPL * railway noise	0.0001 (0.0037)	0.0151	0.9880
Maximum SPL * aircraft noise	−0.0138 (0.0040)	−3.4148	<0.001
Tr	0.0161 (0.0043)	3.7408	<0.001
Noise duration	0.0009 (0.0009)	1.0081	0.3130
Railway noise (indicator)	0.0752 (0.2133)	0.3525	0.7240
Aircraft noise (indicator)	0.5769 (0.2358)	2.4463	0.0144
Noise-free interval	0.0095 (0.0007)	13.24	<0.001
Elapsed sleep time	0.0007 (0.0001)	10.78	<0.001
Elapsed time in same sleep stage	−0.0085 (0.0007)	−12.16	<0.001
Noise number	0.0011 (0.0018)	0.62	0.5330
Maximum SPL * noise number	−0.0001 (0.0000)	−1.88	0.0608
Number of noises per night	−0.0025 (0.0004)	−6.28	<0.001
Sleep stage S3	−0.6396 (0.0399)	−16.04	<0.001
Sleep stage S4	−0.6927 (0.0531)	−13.06	<0.001
REM sleep	−0.2155 (0.0227)	−9.49	<0.001
Age	0.0013 (0.0029)	0.46	0.6430
IfADo study	0.4164 (0.2514)	1.6561	0.0977
STRAIN study	−0.1331 (0.2711)	−0.4910	0.6230
Maximum SPL * IfADo study	−0.0070 (0.0040)	−1.7509	0.0800
Maximum SPL * STRAIN study	0.0074 (0.0044)	1.6779	0.0934

Maximum SPL = maximum A-weighted sound pressure level (SPL) of noise events [dB] from the three major traffic noise sources aircraft, road, and railway traffic; Tr = steepest slope of the event curve as rise time of the maximum A-weighted SPL of a noise event [dB/s]; noise duration = duration of a noise event [s]; noise-free interval = noise-free interval between two noise events [min]; elapsed sleep time = time that participants spent asleep before onset of a noise event [epochs]; elapsed time in sleep stages = cumulative time that participants spent in a certain sleep stage before onset of a noise event [epochs]; noise number = current number of a noise event (noise events were counted consecutively); number of noise events per night = total number of noise events per night; sleep stage S3 + S4 = deep sleep; REM sleep = rapid eye movement sleep; age = age of participants at enrollment [years]. Reference categories: Road traffic noise, AIRORA study.
